# Altered expression, but small contribution, of the histone demethylase KDM6A in obstructive uropathy in mice

**DOI:** 10.1242/dmm.049991

**Published:** 2023-09-01

**Authors:** Lisa Y. Q. Hong, Emily S. H. Yeung, Duc Tin Tran, Veera Ganesh Yerra, Harmandeep Kaur, M. D. Golam Kabir, Suzanne L. Advani, Youan Liu, Sri Nagarjun Batchu, Andrew Advani

**Affiliations:** Keenan Research Centre for Biomedical Science and Li Ka Shing Knowledge Institute, St. Michael's Hospital, Toronto, Ontario M5B 1T8, Canada

**Keywords:** Epigenetic, Histone modification, KDM6A, Inflammation, Tubule cell, PPAR, Unilateral ureteral obstruction

## Abstract

Epigenetic processes have emerged as important modulators of kidney health and disease. Here, we studied the role of KDM6A (a histone demethylase that escapes X-chromosome inactivation) in kidney tubule epithelial cells. We initially observed an increase in tubule cell *Kdm6a* mRNA in male mice with unilateral ureteral obstruction (UUO). However, tubule cell knockout of KDM6A had relatively minor consequences, characterized by a small reduction in apoptosis, increase in inflammation and downregulation of the peroxisome proliferator-activated receptor (PPAR) signaling pathway. In proximal tubule lineage HK-2 cells, KDM6A knockdown decreased PPARγ coactivator-1α (PGC-1α) protein levels and mRNA levels of the encoding gene, *PPARGC1A*. Tubule cell *Kdm6a* mRNA levels were approximately 2-fold higher in female mice than in male mice, both under sham and UUO conditions. However, kidney fibrosis after UUO was similar in both sexes. The findings demonstrate *Kdm6a* to be a dynamically regulated gene in the kidney tubule, varying in expression levels by sex and in response to injury. Despite the context-dependent variation in *Kdm6a* expression, knockout of tubule cell KDM6A has subtle (albeit non-negligible) effects in the adult kidney, at least in males.

## Teaser

**Summary:** Tubule cell KDM6A expression is increased from a sex-determined baseline in obstructive uropathy. However, knockout of KDM6A in male mice causes only subtle changes, characterized by augmented inflammation.

## INTRODUCTION

Over recent years, epigenetic processes have been widely accepted as playing important roles in the pathogenesis of kidney disease (Dwivedi et al., 2011; Guo et al., 2019; Wen et al., 2022). These epigenetic processes may exacerbate kidney injury, they may attenuate it, or they may be dysregulated in disease without contributing to its pathogenesis. For instance, we previously demonstrated that deletion of the histone methyltransferase enhancer of zeste homolog 2 (EZH2) from podocytes augments glomerular disease (Majumder et al., 2018). By contrast, we found that the EZH2-approximating long non-coding RNA, HOTAIR, is dysregulated in diabetic kidney disease but inconsequential to its pathogenesis (Majumder et al., 2019). EZH2 is a histone methyltransferase that catalyzes the trimethylation of lysine residue 27 on histone protein H3, a marker of gene repression (Cao et al., 2002). This same epigenetic mark can be erased by lysine-specific demethylase 6 (KDM6)A and KDM6B (Agger et al., 2007; Lan et al., 2007; Lee et al., 2007), and we previously reported that inhibition of KDM6 demethylases attenuated glomerular disease caused by diabetes, adriamycin nephrotoxicity or renal mass ablation (Majumder et al., 2018), a finding substantiated elsewhere (Lin et al., 2019). The biological functions of KDM6A, however, extend beyond its actions in glomerular podocytes. For instance, mutations in *KDM6A* are responsible for Kabuki syndrome (Bögershausen et al., 2016), a rare multisystem disorder associated with congenital abnormalities of the kidney and urinary tract, and they have also been linked to bladder and kidney cancer (Dalgliesh et al., 2010; Nickerson et al., 2014). Thus, the actions of KDM6A in cells within the kidney and urinary tract, other than podocytes, are likely to be important, but these actions remain largely unresolved.

*KDM6A* [also called ubiquitously transcribed tetratricopeptide repeat on chromosome X (*UTX*)] is expressed on the X chromosome. In women, ∼15% of X-linked genes escape inactivation, and in female mice this number is ∼3% (Berletch et al., 2011). *KDM6A* is one of the minority of genes that does escape X inactivation (Greenfield et al., 1998). Males, though, carry the Y-linked homolog, *UTY* (also called *KDM6C*), which bears 88% homology to *KDM6A*, although possesses minimal H3K27-demethylating activity (Hong et al., 2007; Lan et al., 2007; Walport et al., 2014). KDM6A is a Jumonji C (JmjC) domain-containing family member that exerts its lysine-demethylating effects through its JmjC catalytic domain (Tsukada et al., 2006), which also has 84% sequence similarity to that of KDM6B (Hong et al., 2007). However, KDM6A also possesses six tetratricopeptide repeat (TPR) domains that can mediate protein interactions, whereas KDM6B does not (Hong et al., 2007). Accordingly, KDM6A can have both enzymatic (lysine-demethylating) and non-enzymatic effects, and its enzymatic effects can either overlap with those of other H3K27me3 demethylating enzymes or they can be unique to KDM6A (Lan et al., 2007; Ntziachristos et al., 2014).

Here, we set out to further illuminate the roles of KDM6A in the kidney. Cognizant that advances in chronic kidney disease (CKD) therapeutics have shifted the spotlight towards damage of kidney tubules as being the primary force in CKD progression (Gilbert, 2017; Heerspink et al., 2020; Liu et al., 2018), we explored the expression and actions of KDM6A in tubule epithelial cells.

## RESULTS

### *Kdm6a* is upregulated in kidney tubule epithelial cells of male mice with unilateral ureteral obstruction (UUO)

**Fig. 1.**
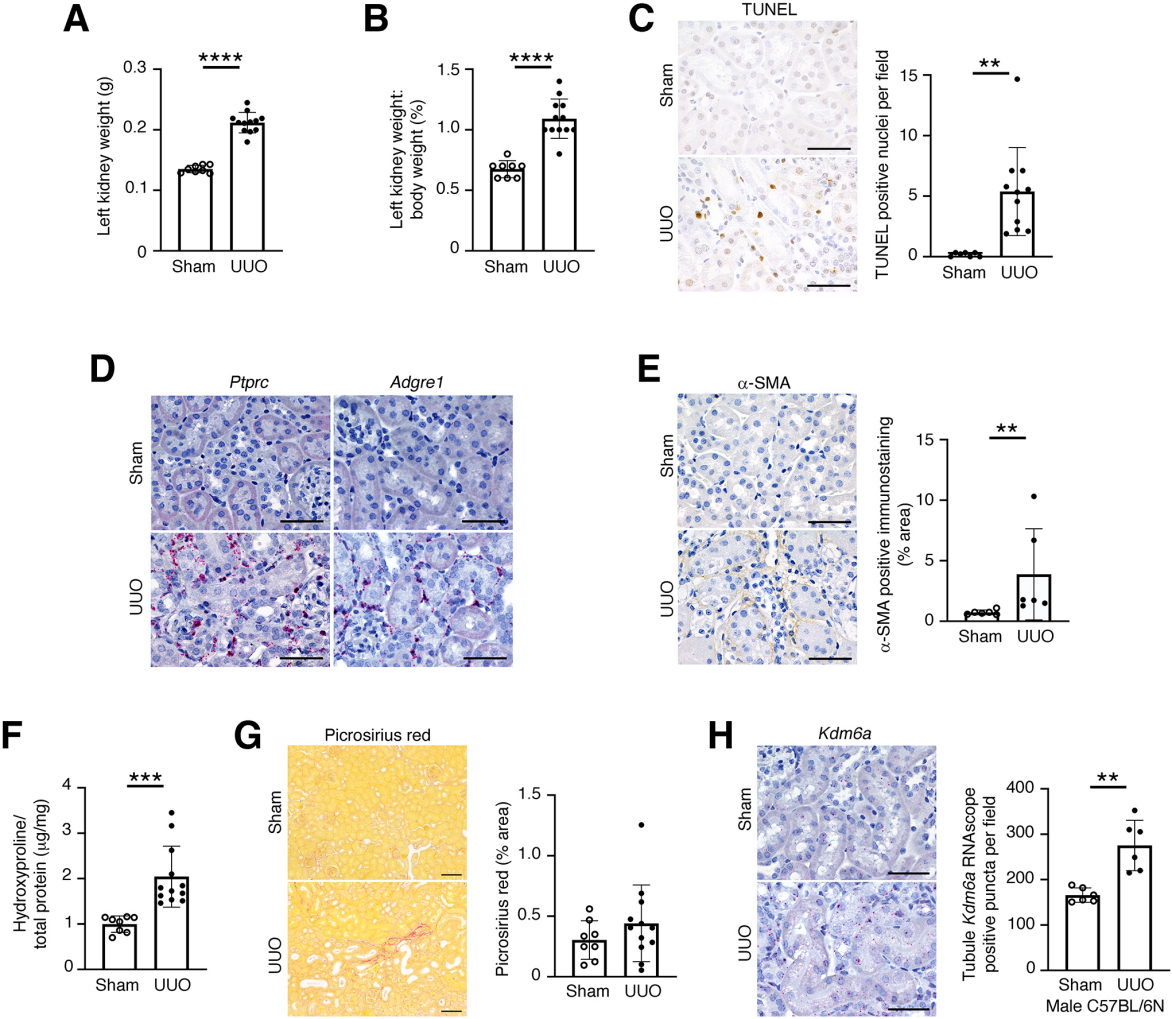
***Kdm6a* is upregulated in tubule cells of mice 7 days after unilateral ureteral obstruction (UUO).** (A,B) Left kidney weight (A) and left kidney weight:body weight ratio (B) in male C57BL/6N mice 7 days after sham surgery (A; *n*=8) or UUO surgery (B; *n*=12). (C) Representative TUNEL staining (left) and quantitation of TUNEL^+^ nuclei per 100× field (sham, *n*=7; UUO, *n*=11) (right). Original magnification 400×. Scale bars: 50 µm. (D) RNAscope *in situ* hybridization for *Ptprc* and *Adgre1* in the kidneys of mice 7 days after sham or UUO surgery. Original magnification 400×. Scale bars: 50 µm. Images are representative of *n*=3/group. (E) Immunohistochemistry for α-smooth muscle actin (α-SMA) (left) and quantitation of α-SMA immunopositivity in kidney sections of mice 7 days after sham or UUO surgery (*n*=6/group) (right). Original magnification 400×. Scale bars: 50 µm. (F) Kidney hydroxyproline content (sham, *n*=8; UUO, *n*=12). (G) Representative images of Picrosirius Red-stained kidney sections (left) and quantitation of interstitial Picrosirius Red staining in sham-operated (*n*=8) and UUO (*n*=12) mouse kidneys (right). Original magnification 100×. Scale bars: 100 µm. (H) RNAscope *in situ* hybridization for *Kdm6a* in the kidneys of male mice 7 days after sham or UUO surgery (left) and quantitation of tubule *Kdm6a* RNAscope puncta (red) (*n*=6/group) (right). Original magnification 400×. Scale bars: 50 µm. Values are mean±s.d. **P<0.01, ***P<0.001, ****P<0.0001 by unpaired two-tailed *t*-test with Welch's correction (A,B,F,H) or unpaired two-tailed Mann–Whitney test (C,E).

**Fig. 2.**
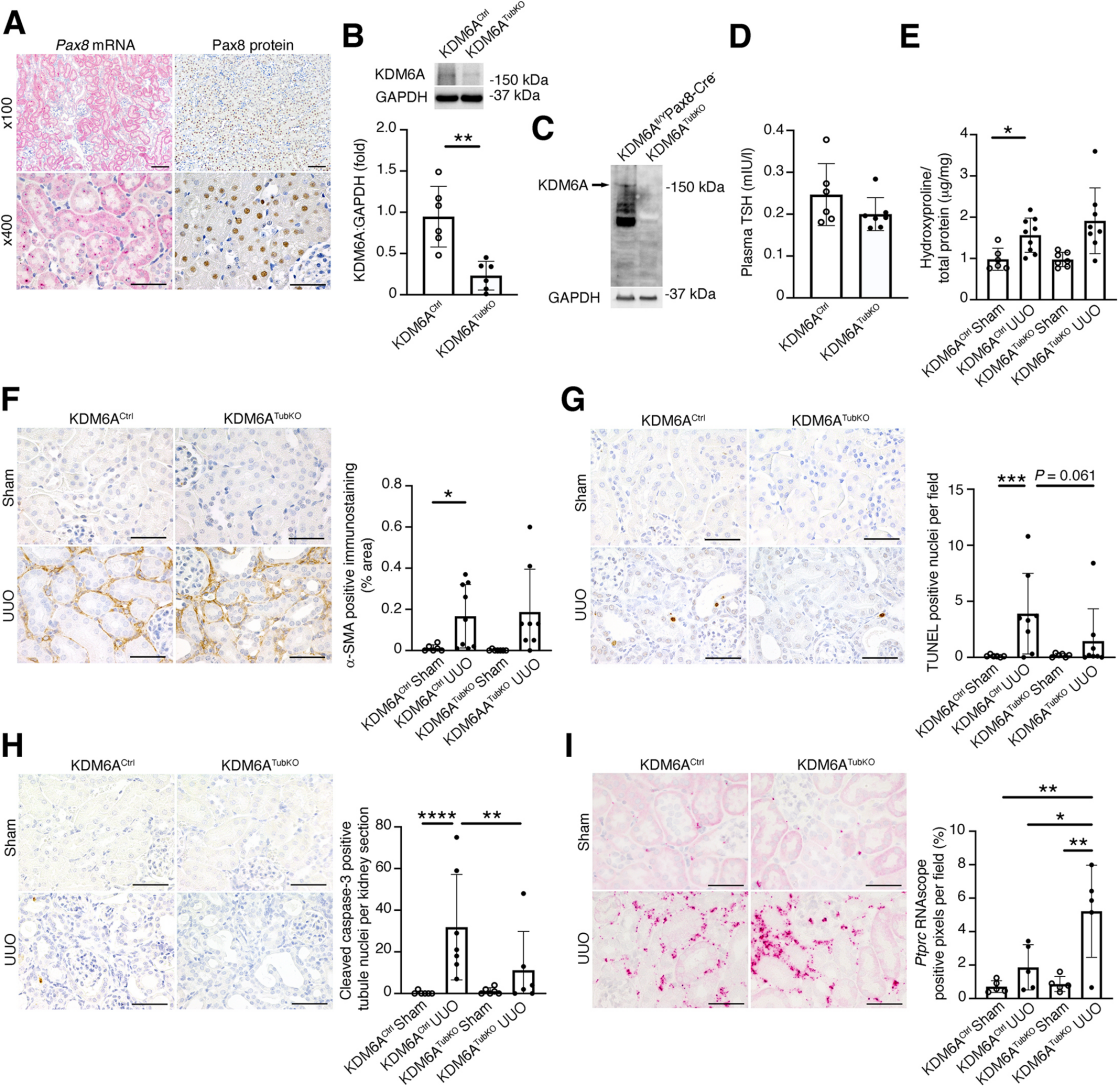
**Knockout of *Kdm6a* from tubule epithelial cells increases inflammatory cell infiltration in male mice 7 days after UUO.** (A) RNAscope *in situ* hybridization and immunohistochemistry for Pax8 in mouse kidneys. Top row, scale bars: 50 µm; bottom row, scale bars: 100 µm. Images are representative of *n*=5 for *in situ* hybridization and *n*=6 for immunohistochemistry. (B) Immunoblotting for KDM6A in kidney homogenates from KDM6A^Ctrl^ mice and KDM6A^TubKO^ mice (*n*=6/group). (C) Immunoblotting for KDM6A in primary tubule epithelial cells from KDM6A^fl/Y^*Pax8-*Cre^−^ and KDM6A^TubKO^ kidneys. Representative of three experiments. (D) Plasma TSH in KDM6A^Ctrl^ and KDM6A^TubKO^ mice (KDM6A^Ctrl^, *n*=6; KDM6A^TubKO^, *n*=7). *P*=0.17 by unpaired two-tailed Student's *t*-test. (E) Kidney hydroxyproline content (KDM6A^Ctrl^ sham, *n*=6; KDM6A^Ctrl^ UUO, *n*=9; KDM6A^TubKO^ sham, *n*=7; KDM6A^TubKO^ UUO, *n*=8). (F) Representative photomicrographs of α-SMA immunostaining (left) and quantitation of α-SMA immunopositivity (KDM6A^Ctrl^ sham, *n*=6; KDM6A^Ctrl^ UUO, *n*=9; KDM6A^TubKO^ sham, *n*=7; KDM6A^TubKO^ UUO, *n*=8) (right). Original magnification 400×. Scale bars: 50 µm. (G) Representative TUNEL staining (left) and quantitation of TUNEL^+^ nuclei per 100× field (KDM6A^Ctrl^ sham, *n*=6; KDM6A^Ctrl^ UUO, *n*=8; KDM6A^TubKO^ sham, *n*=6; KDM6A^TubKO^ UUO, *n*=8) (right). Original magnification 400×. Scale bars: 50 µm. (H) Representative cleaved caspase-3 staining (left) and quantitation of cleaved caspase-3^+^ tubule nuclei per kidney section (KDM6A^Ctrl^ sham, *n*=6; KDM6A^Ctrl^ UUO, *n*=7; KDM6A^TubKO^ sham, *n*=6; KDM6A^TubKO^ UUO, *n*=6) (right). Original magnification 400×. Scale bars: 50 µm. (I) RNAscope *in situ* hybridization for *Ptprc* (left) and quantitation of *Ptprc*^+^ pixels per digitized kidney cross-section (*n*=5/group) (right). Original magnification 400×. Scale bars: 50 µm. Values are mean±s.d. *P<0.05, **P<0.01, ***P<0.001, ****P<0.0001 by two-tailed unpaired Student’s *t*-test (B), or by Brown–Forsythe ANOVA followed by Dunnett's T3 multiple comparisons test (E,F), one-way ANOVA followed by Sidak's post-test of log-transformed data (G,H), or one-way ANOVA followed by Tukey's post-test (I).

**Fig. 3.**
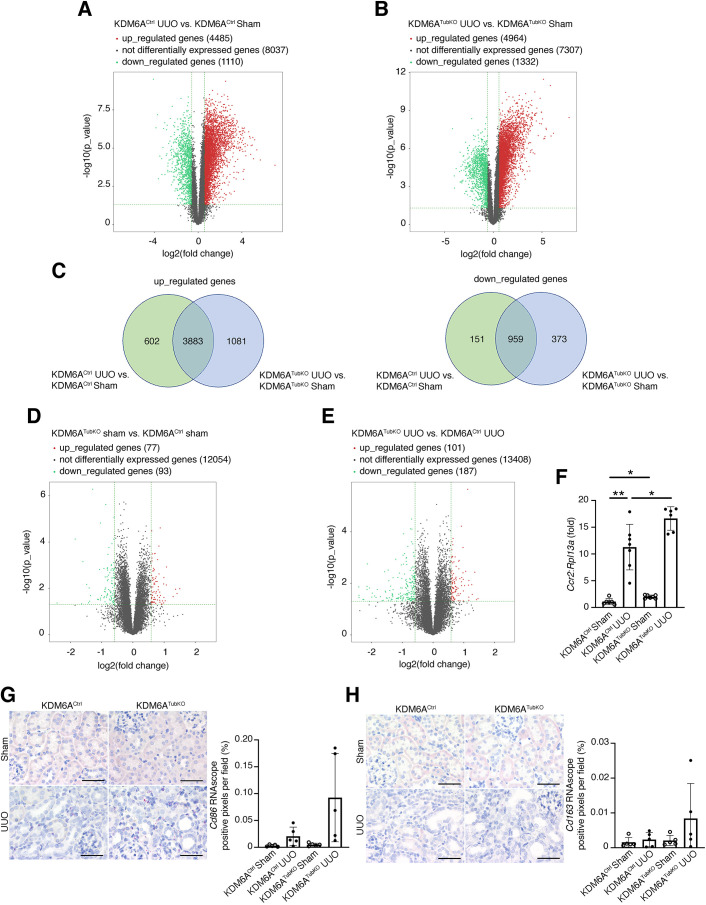
**Transcriptional changes in the kidneys of KDM6A^Ctrl^ and KDM6A^TubKO^ mice after sham or UUO surgery (*n*=5/group).** (A,B) Volcano plots of differentially expressed genes following RNA sequencing of mouse kidney homogenates from KDM6A^Ctrl^ and KDM6A^TubKO^ mice 7 days after sham or UUO surgery. (A) KDM6A^Ctrl^ UUO versus KDM6A^Ctrl^ sham. (B) KDM6A^TubKO^ UUO versus KDM6A^TubKO^ sham. (C) Venn diagrams showing number of shared and unique differentially expressed genes for the comparisons in A and B. (D,E) Volcano plots of differentially expressed genes following RNA sequencing of mouse kidney homogenates from KDM6A^TubKO^ sham versus KDM6A^Ctrl^ sham (D), and KDM6A^TubKO^ UUO versus KDM6A^Ctrl^ UUO (E). Red/green circles in A,B,D,E indicate statistically significant differentially expressed genes with fold change no less than 1.5 and *P*≤0.05 (red, upregulated; green, downregulated). Gray circles indicate genes that are not differentially expressed. (F) Quantitative reverse transcription polymerase chain (qRT-PCR) for *Ccr2* in KDM6A^Ctrl^ and KDM6A^TubKO^ sham-operated and UUO kidneys (KDM6A^Ctrl^ sham, *n*=6; KDM6A^Ctrl^ UUO, *n*=7; KDM6A^TubKO^ sham, *n*=7; KDM6A^TubKO^ UUO, *n*=6). (G,H) RNAscope *in situ* hybridization for *Cd86* (G) and *Cd163* (H) (left) and quantitation of positive pixels per digitized kidney cross section (*n*=5/group) (right). Original magnification 400×. Scale bars: 50 µm. Values are mean±s.d. *P<0.05, **P<0.01 by Brown–Forsythe ANOVA followed by Dunnett's T3 multiple comparisons test.

We began our studies by examining the expression patterns of *Kdm6a* in the kidneys of male mice after UUO, a well-established model of obstructive uropathy that causes inflammation and fibrosis (Chevalier et al., 2009), central determinants of the progression of CKD to end-stage kidney disease (Chung et al., 2019; Ruiz-Ortega et al., 2020). In comparison to sham-operated mice, mice 1 week after UUO exhibited an increase in weight of the obstructed kidney (Fig. 1A,B) that was accompanied by increases in (1) tubule programmed cell death as determined by terminal deoxynucleotidyl transferase dUTP nick end labeling (TUNEL) staining (Fig. 1C); (2) inflammatory cell accumulation within the kidney tubulointerstitium as determined by RNAscope *in situ* hybridization for protein tyrosine phosphatase receptor, type C (*Ptprc*; the gene that encodes the pan-leukocyte marker CD45) and adhesion G protein-coupled receptor E1 (*Adgre1*; the gene that encodes the macrophage marker F4/80) (Fig. 1D); (3) interstitial α-smooth muscle actin (α-SMA; also called ACTA2) immunostaining (Fig. 1E), indicative of fibroblast activation; and (4) kidney hydroxyproline content (Fig. 1F), indicative of increased collagen production. At the timepoint studied (7 days), although there were focal areas of cortical scarring evident in UUO kidneys, this did not result in an increase in Picrosirius Red staining across whole-kidney cross-sections, detectable by image analysis (Fig. 1G). By RNAscope *in situ* hybridization, *Kdm6a* transcripts were observed in several kidney cell types, including glomerular cells, parietal epithelial cells, tubule cells and interstitial cells (Fig. 1H). Manual counting of RNAscope puncta revealed an ∼67% increase in the number of labeled *Kdm6a* transcripts in tubule epithelial cells of UUO kidneys in comparison to those of kidneys of sham-operated mice (Fig. 1H).

### Knockout of *Kdm6a* from tubule epithelial cells does not affect kidney fibrosis but is associated with attenuated tubule apoptosis and increased kidney inflammation

To determine whether tubule-expressed *Kdm6a* contributes to kidney damage following obstructive injury, we generated tubule- specific KDM6A knockout mice. We did this by breeding *Kdm6a*^fl/fl^ mice (Manna et al., 2015) with *Pax8*-Cre^+^ mice that express Cre recombinase localized to the tubule epithelium in the kidney (Bouchard et al., 2004). Because the *Kdm6a* gene is X linked, hemizygous *Pax8*-Cre^+^*Kdma6a*^fl/Y^ were designated as tubule-specific KDM6A knockouts (henceforth KDM6A^TubKO^) and male *Pax8*-Cre^+^ mice were used as controls (henceforth KDM6A^Ctrl^). RNAscope *in situ* hybridization and immunohistochemistry of normal mouse kidneys demonstrated Pax8 expression in the nuclei of tubule epithelial cells throughout the length of the nephron (Fig. 2A), and immunoblotting revealed an ∼75% decrease in KDM6A protein in the kidneys of KDM6A^TubKO^ mice, together with the absence of KDM6A protein from primary tubule epithelial cells (Fig. 2B,C). Given that Pax8 is also expressed in the thyroid gland (Plachov et al., 1990), to determine whether KDM6A knockout affects thyroid function, we measured plasma thyroid stimulating hormone (TSH) levels, observing no difference between KDM6A^Ctrl^ and KDM6A^TubKO^ mice (Fig. 2D). KDM6A^Ctrl^ and KDM6A^TubKO^ mice were then subjected to sham or UUO surgery and were followed for 7 days. Enlargement of the obstructed kidney was equivalent in KDM6A^TubKO^ UUO mice and KDM6A^Ctrl^ UUO mice (Table S1). Similarly, kidney hydroxyproline content (Fig. 2E) and α-SMA immunopositivity (Fig. 2F) were each equivalently increased in KDM6A^TubKO^ UUO mice and KDM6A^Ctrl^ UUO mice. By contrast, tubule cell TUNEL positivity was increased in KDM6A^Ctrl^ UUO kidneys and tended to be lower in KDM6A^TubKO^ UUO kidneys, although this difference was not statistically significant (Fig. 2G). Cleaved caspase-3 immunopositivity was increased in KDM6A^Ctrl^ UUO kidneys and was significantly lower in KDM6A^TubKO^ UUO kidneys (Fig. 2H). By RNAscope *in situ* hybridization, we observed an increase in *Ptprc* RNAscope puncta in KDM6A^TubKO^ UUO kidneys relative to those in KDM6A^Ctrl^ UUO kidneys (Fig. 2I), suggestive of enhanced kidney inflammation with tubule cell *Kdm6a* knockout.

### Effects of tubule-specific knockout of *Kdm6a* on sham and UUO kidney transcriptomes

Next, to determine the effects of tubule cell knockout of *Kdm6a* on transcriptional changes in sham mouse kidneys and UUO mouse kidneys, we performed next-generation sequencing of RNA isolated from the kidneys of sham-operated and UUO KDM6A^Ctrl^ and KDM6A^TubKO^ mice. Among KDM6A^Ctrl^ mice, of 13,632 gene counts, 4485 (32.9%) genes were upregulated and 1110 (8.1%) were downregulated (fold change ≥1.5, *P*<0.05) in UUO kidneys in comparison to sham-operated kidneys (Fig. 3A); in KDM6A^TubKO^ mice, of 13,603 gene counts, 4964 genes (36.5%) were upregulated and 1332 (9.8%) were downregulated in UUO kidneys in comparison to sham-operated kidneys (Fig. 3B). Fig. 3C shows the number of overlapping and unique differentially expressed genes in the KDM6A^Ctrl^ and KDM6A^TubKO^ UUO mice in comparison to their respective control groups. In contrast to the difference between sham and UUO, as illustrated by the volcano plots in Fig. 3D and E, tubule cell knockout of *Kdm6a* had comparatively little effect on kidney transcriptional changes, either in sham-operated mice (Fig. 3D) or in UUO mice (Fig. 3E).

Tables 1 and 2 show the top 20 upregulated (Table 1) and downregulated (Table 2) genes in UUO and sham kidneys from KDM6A^Ctrl^ and KDM6A^TubKO^ mice. The most upregulated gene in UUO mice was *Havcr1*, the gene encoding kidney injury molecule-1 (KIM-1), which was upregulated 131-fold in KDM6A^Ctrl^ UUO kidneys in comparison to KDM6A^Ctrl^ sham-operated kidneys and 220-fold in KDM6A^TubKO^ UUO kidneys in comparison to KDM6A^TubKO^ sham-operated kidneys (Table 1). Similarly, *Lcn2*, the gene encoding neutrophil gelatinase-associated lipocalin (NGAL), was upregulated 34-fold in KDM6A^Ctrl^ UUO kidneys and 73-fold in KDM6A^TubKO^ UUO kidneys (Table 1). A full list of differentially expressed genes is provided in Dataset 1.

**Table 1.**
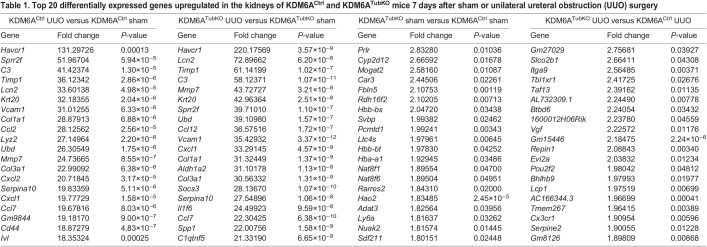
Top 20 differentially expressed genes upregulated in the kidneys of KDM6A^Ctrl^ and KDM6A^TubKO^ mice 7 days after sham or unilateral ureteral obstruction (UUO) surgery

**Table 2.**
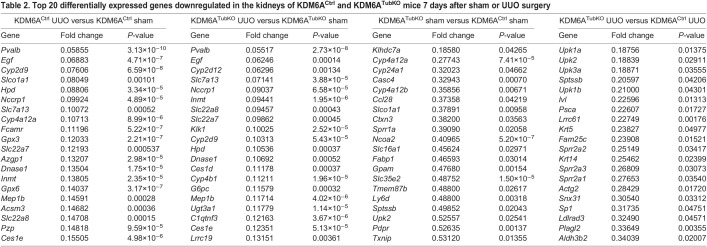
Top 20 differentially expressed genes downregulated in the kidneys of KDM6A^Ctrl^ and KDM6A^TubKO^ mice 7 days after sham or UUO surgery

Although less than 2% of genes were differentially expressed in either sham or UUO KDM6A^TubKO^ kidneys in comparison to KDM6A^Ctrl^ kidneys, several pro-inflammatory cytokines were differentially upregulated in KDM6A^TubKO^ UUO kidneys (versus KDM6A^Ctrl^ UUO kidneys), including *Cx3cr1* (fold change=1.91, *P*=0.0060), *Ccl12* (fold change=1.79, *P*=0.0295), *Ccr2* (fold change=1.79, *P*=0.0027) and *Cxcl14* (fold change=1.76, *P*=0.0254). Given the accumulation of macrophages in UUO kidneys (Fig. 1D) and the importance of macrophage recruitment in CCL2/CCR2 signaling between injured tubule cells and pro-inflammatory macrophages (Xu et al., 2019), we sought to validate our RNA-sequencing findings by performing quantitative reverse transcription polymerase chain reaction (qRT-PCR) for *Ccr2*. Here, we observed an expected increase in *Ccr2* mRNA in UUO mice, with a further incremental increase in *Ccr2* mRNA levels with KDM6A knockout (Fig. 3F), as was observed by RNA sequencing. We next set out to determine whether the macrophages that accumulate 7 days after UUO in KDM6A^Ctrl^ and KDM6A^TubKO^ kidneys are predominantly pro-inflammatory ‘M1-like’ or reparative ‘M2-like’. To do this, we performed RNAscope *in situ* hybridization for the M1 marker *Cd86* and the M2 marker *Cd163* (Fig. 3G,H). In these experiments, we observed that most infiltrating macrophages in mouse kidneys 7 days after UUO are pro-inflammatory M1-like *Cd86*^+^ cells (Fig. 3G,H). Although *Cd86*^+^ puncta and *Cd163*^+^ puncta were numerically increased in KDM6A^TubKO^ UUO kidneys, this did not reach statistical significance in multiple group comparisons (Fig. 3G,H).

### Kyoto Encyclopedia of Genes and Genomes (KEGG) pathway analysis reveals downregulation of peroxisome proliferator-activated receptor (PPAR) signaling pathways with tubule cell *Kdm6a* knockout, associated with increased immune pathway activation

To identify which molecular interaction/reaction networks are specifically affected by tubule cell *Kdm6a* knockout, we performed KEGG pathway analysis of the differentially expressed genes. The top five upregulated and downregulated pathways are shown in Tables S2-S5, and a full list of dysregulated pathways and the differentially expressed genes contributing to these pathways is provided in Dataset 2. Because KDM6A is best understood to mediate gene activation (Agger et al., 2007; Lan et al., 2007; Lee et al., 2007), we reasoned that *Kdm6a* knockout would be most likely to directly affect pathway downregulation. Accordingly, we focused our attention on pathways that were downregulated in KDM6A^TubKO^ sham-operated mice and KDM6A^TubKO^ UUO mice in comparison to KDM6A^Ctrl^ sham-operated and KDM6A^Ctrl^ UUO mice, respectively (Tables S4 and S5). Mmu03320 PPAR_signaling_pathway was the most downregulated pathway in the KEGG pathway comparison of KDM6A^TubKO^ and KDM6A^Ctrl^ kidneys in sham-operated mice (Fig. S1A) and the second most downregulated pathway in the comparison of KDM6A^TubKO^ and KDM6A^Ctrl^ UUO kidneys (Fig. S1B). The most upregulated KEGG pathway in KDM6A^TubKO^ UUO kidneys (in comparison to KDM6A^Ctrl^ UUO kidneys) was mmu04061 Viral_protein_interaction_with_cytokine_and_cytokine_receptor (Fig. S1C), indicative of augmented tissue inflammation with *Kdm6a* knockout from tubule epithelial cells.

### Knockdown of KDM6A downregulates peroxisome proliferator-activated receptor gamma coactivator 1-alpha (PGC-1α) in HK-2 cells

**Fig. 4.**
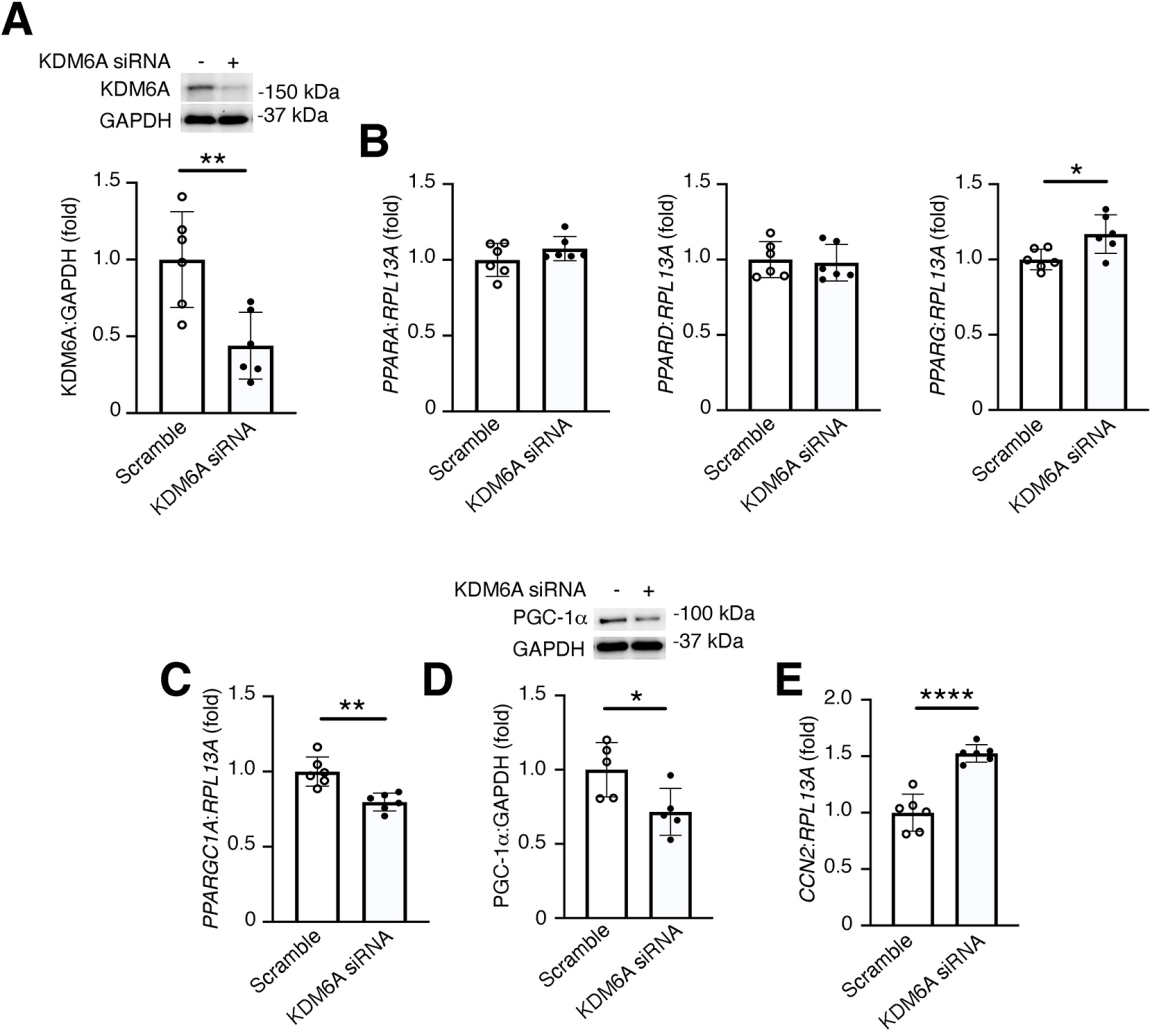
**Knockdown of KDM6A downregulates PGC-1α in HK-2 cells.** (A) Immunoblotting of HK-2 cells transfected with scramble or KDM6A siRNA for 48 h (*n*=6/condition). (B) qRT-PCR for *PPARA* (left), *PPARD* (middle) and *PPARG* (right) in HK-2 cells transfected with scramble or KDM6A siRNA for 48 h (*n*=6/condition). (C,D) qRT-PCR (C; *n*=6/condition) and immunoblotting (D; *n*=5/condition) for *PPARGC1A/*PGC1-α in HK-2 cells transfected with scramble or KDM6A siRNA for 48 h. (E) qRT-PCR for *CCN2* in HK-2 cells transfected with scramble or KDM6A siRNA for 48 h (*n*=6/condition). Values are mean±s.d. *P<0.05, **P<0.01, ****P<0.0001 by unpaired two-tailed Student’s *t*-test.

PPAR signaling can have both anti-inflammatory (Straus and Glass, 2007) and pro-apoptotic (Gao et al., 2015) effects. Given the histological evidence we had seen of decreased tubule cell apoptosis (Fig. 2G,H) and increased kidney inflammation (Fig. 2I, Fig. 3F,G) with KDM6A knockout, we were interested to explore further whether KDM6A regulates PPARs in tubule epithelial cells. For these experiments, we used the human proximal tubule HK-2 cell line to circumvent limitations imposed by bulk tissue analysis of mixed cell populations. As expected, transfection with *KDM6A* short interfering RNA (siRNA) caused a significant reduction in KDM6A protein levels in HK-2 cells (Fig. 4A). However, this did not result in a significant change in *PPARA* or *PPARD* mRNA levels, whereas *PPARG* mRNA levels were unexpectedly increased (Fig. 4B). We speculated, therefore, that KDM6A can regulate PPAR signaling by altering the expression of one of the PPAR binding partners that regulates gene transcription in response to PPAR signaling. Unlike PPAR isoforms themselves, we observed that mRNA levels of *PPARGC1A* (the gene encoding PGC-1α) were significantly reduced in HK-2 cells with KDM6A knockdown (Fig. 4C), accompanied by a reduction in PGC-1α protein levels (Fig. 4D) and a significant upregulation in the expression of the pro-fibrotic gene *CCN2* (Fig. 4E).

### Despite higher tubule cell *Kdm6a* mRNA levels in females than in males, kidney fibrosis after UUO is equivalent in males and females

**Fig. 5.**
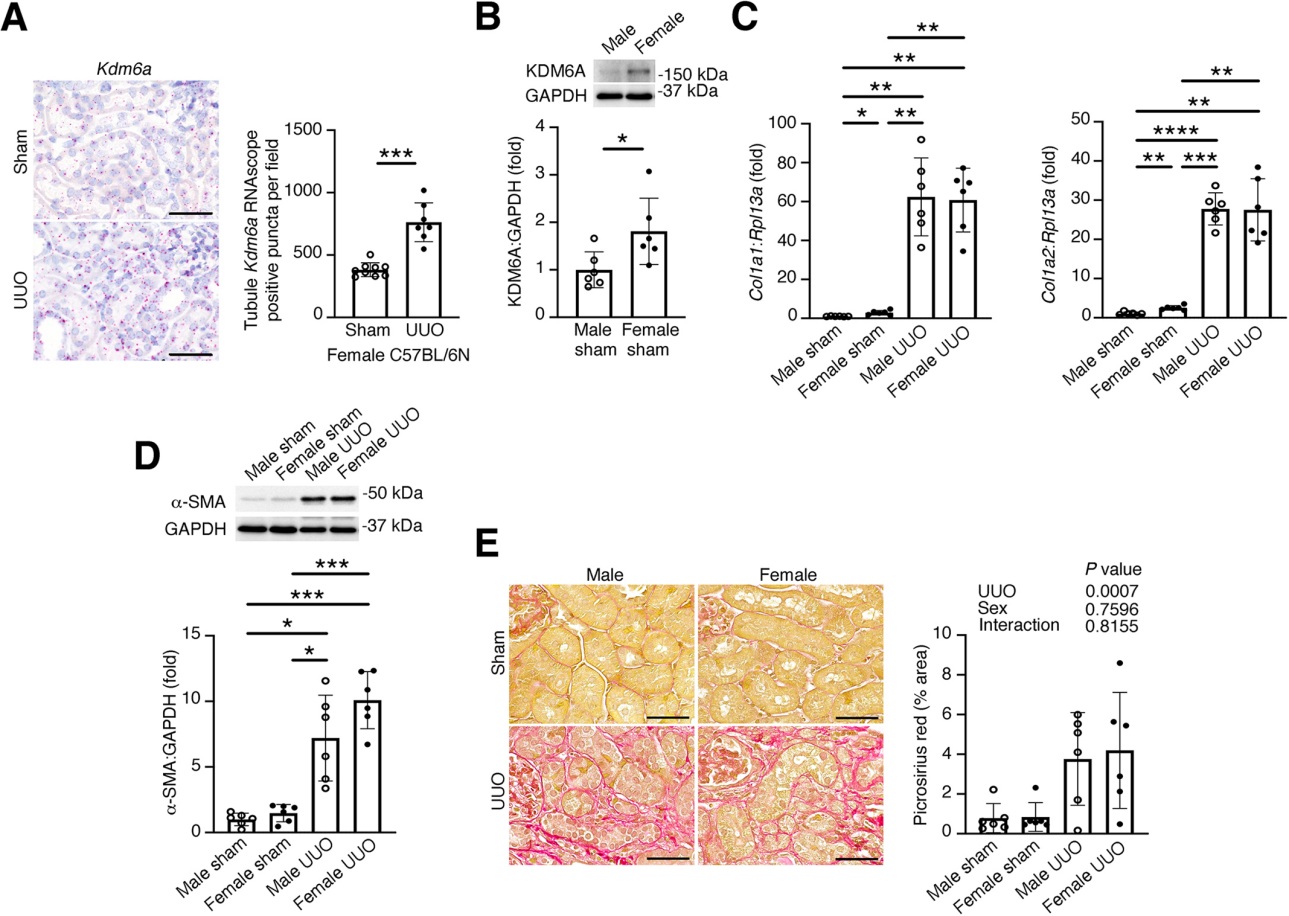
**Tubule cell *Kdm6a* is increased in female mice, but kidney fibrosis is similar in males and females 14 days after UUO.** (A) RNAscope *in situ* hybridization for *Kdm6a* in the kidneys of female C67BL/6N mice 7 days after sham or UUO surgery (left) and quantitation of tubule *Kdm6a* RNAscope puncta (red) (sham, *n*=9; UUO, *n*=7) (right). Original magnification 400×. Scale bars: 50 µm. (B) Immunoblotting of kidney homogenates of sham-operated male and female C57BL/6N mice for KDM6A (*n*=6/group). (C) qRT-PCR for *Col1a1* (left) and *Col1a2* (right) in the kidneys of male and female C57BL/6N mice 14 days after sham or UUO surgery (*n*=6/group). (D) Immunoblotting for α-SMA in kidney homogenates of male and female C57BL/6N mice 14 days after sham or UUO surgery (*n*=6/group). (E) Representative images of Picrosirius Red-stained kidney sections (left) and quantitation of interstitial Picrosirius Red staining of kidney sections from male and female C57BL/6N mice 14 days after sham or UUO surgery (*n*=6/group) (right). Original magnification 400×. Scale bars: 50 µm. Values are mean±s.d. *P<0.05, **P<0.01, ***P<0.001, ****P<0.0001 by unpaired two-tailed *t*-test with Welch's correction (A,B), Brown–Forsythe ANOVA followed by Dunnett's T3 multiple comparisons test (C,D) or two-way ANOVA (E).

Lastly, we reflected on our experiments and that our studies, to date, had been performed in male mice and in a cell line that had been derived from a male donor. Cognizant that KDM6A escapes X-chromosome inactivation (Greenfield et al., 1998), we studied a further cohort of female C57BL/6N mice 7 days after sham or UUO surgery. We used the same RNAscope *in situ* hybridization approach to quantify tubule cell *Kdm6a* transcripts as we had used in our initial experiments in male mice (Fig. 1H). Taking this approach, we observed that tubule epithelial cell *Kdm6a* levels in sham-operated female mice were approximately double those in male mice and, like in males, they were increased after UUO (Fig. 1H and Fig. 5A). Similarly, immunoblotting kidney homogenates of male and female mice revealed increased KDM6A protein abundance in female mice in comparison to male mice (Fig. 5B). Given the increased inflammation in male KDM6A^TubKO^ UUO kidneys (Fig. 2I, Fig. 3F,G), and the downregulation of PGC-1α and upregulation of *CCN2* in male HK-2 cells with KDM6A knockdown (Fig. 4C-E), we speculated that if heightened *Kdm6a* mRNA levels in female mice substantially compensate for kidney tubule injury this would be manifested by an attenuation in kidney fibrosis in comparison to that in male mice. Accordingly, we subjected male and female C57BL/6N mice to sham or UUO surgery, and we followed the mice for 14 days (Table S6), extending the period of follow up because of the previously observed absence of difference in Picrosirius Red staining 7 days after UUO (Fig. 1G). *Col1a1* and *Col1a2* mRNA levels were marginally, albeit significantly, higher in the kidneys of sham-operated female mice than in those of sham-operated male mice (Fig. 5C). However, *Col1a1* and *Col1a2* mRNA was increased equivalently (∼30- to 60-fold) in males and females after UUO (Fig. 5C), as was α-SMA protein abundance as determined by immunoblotting (Fig. 5D). Similarly, Picrosirius Red staining was increased 14 days after UUO and was unaffected by mouse sex (Fig. 5E).

## DISCUSSION

In the present study, we explored the expression patterns of *Kdm6a* in kidney tubule epithelial cells and the effects of *Kdm6a* knockout from tubule epithelial cells in mice under sham-operated conditions and with obstructive uropathy caused by UUO. Knockout of *Kdm6a* from tubule cells resulted in relatively small, albeit detectable, transcriptional and histological changes in male mice with UUO, including diminished tubule cell apoptosis and augmented inflammation; in HK-2 cells, KDM6A knockdown decreased PGC-1α levels. Consistent with X-chromosome inactivation escape, tubule cell *Kdm6a* transcript abundance was higher in female mice than in male mice, with *Kdm6a* mRNA levels increasing with UUO proportionately in males and females from their baseline sex-determined levels. Despite heightened *Kdm6a* in female mice, kidney fibrosis occurred similarly in females and males following UUO. Collectively, the findings illustrate the relatively subtle consequences of deletion of a single histone demethylase from a single cell type in the kidney and that ostensibly ‘epigenetic’ enzymes can be dynamically regulated in the kidney without being causatively implicated in disease pathogenesis.

We initially studied mice 7 days after UUO, at a point in time characterized by tubule programmed cell death, kidney inflammation and heightened collagen production, but prior to the development of extensive interstitial fibrosis likely to represent irreversible parenchymal loss. At this point in time, we observed upregulation of tubule cell *Kdm6a* levels, like that previously observed in the glomeruli, podocytes, mesangial cells or tubule cells of mice and humans with diabetes or focal segmental glomerulosclerosis (Chen et al., 2019; Lin et al., 2019; Majumder et al., 2018). The causes of KDM6A upregulation in kidney disease are uncertain. Seven days after UUO, kidneys exhibit evidence of activation of epidermal growth factor receptor (EGFR) signaling (Liu et al., 2011), and EGFR has been reported to transcriptionally upregulate *Kdm6a* (Zhou et al., 2022). Conversely, however, KDM6A has been reported to be oxygen sensitive, with hypoxia decreasing KDM6A activity (Chakraborty et al., 2019). Renal tubule hypoxia precedes tubulointerstitial fibrosis in UUO kidneys (Higgins et al., 2007), and thus increases in *Kdm6a* mRNA levels could alternatively represent compensation for diminished KDM6A enzymatic activity. In addition to an upregulation in tubule *Kdm6a* with UUO, we observed higher *Kdm6a* mRNA levels in the tubule cells of female mice in comparison to those of male mice, both under sham-operated conditions and after UUO. Interestingly, despite the well-characterized escape of KDM6A from X-chromosome inactivation in mice and humans (Greenfield et al., 1998), there is surprisingly little published literature describing sex-dependent differences in KDM6A in the kidney. In our previous work, we did not observe a difference in human podocyte KDM6A protein levels in male and female human kidney tissue, albeit by immunohistochemistry and in a small number of tissue samples (Majumder et al., 2018). Similarly, sex differences in KDM6A expression implicating KDM6A upregulation in renal cell carcinoma have not been described in the literature (Shen et al., 2012; Wang et al., 2016). In the present study, despite higher *Kdm6a* levels in female kidneys than in male kidneys, and upregulation of these levels with UUO in both sexes, the fibrogenic response to UUO was similar. Thus, tubule cell *Kdm6a* varies, increasing in response to injury from a sex-determined baseline. However, higher levels do not necessarily imply a role for *Kdm6a* in disease pathogenesis.

Although, in comparison to sham-operated male mice as a point of reference, higher tubule cell *Kdm6a* levels do not indicate a major role for KDM6A in kidney disease pathogenesis, this does not exclude an influence of constitutively expressed KDM6A on the natural history of disease. To explore this possibility, we studied KDM6A^TubKO^ mice. We did this by breeding mice with LoxP sites placed around the JmjC domain-encoding exon of the *Kdm6a* gene (Manna et al., 2015) with tubule-specific *Pax8*-Cre^+^ mice (Bouchard et al., 2004), with our histological survey demonstrating Pax8 expression in normal mice extending throughout the length of the kidney tubule. KDM6A^TubKO^ mice were viable and fertile, without overt phenotypic abnormality detected under sham-operated conditions, indicating a non-essential role of KDM6A in renal homeostasis. Although we observed neither exacerbation nor attenuation of kidney fibrosis 7 days after UUO in KDM6A^TubKO^ mice, the deletion of *Kdm6a* from tubule epithelial cells was not entirely without consequence. Most notably, we observed a small, but consistent, increase in kidney inflammation as determined by RNA sequencing, qRT-PCR and RNAscope *in situ* hybridization. This was accompanied by an apparently paradoxical reduction in tubule cell apoptosis.

In a search for a unifying explanation for the modest histological and transcriptional consequences of tubule cell *Kdm6a* knockout, we focused on the PPAR signaling downregulation observed by KEGG pathway analysis, and we shifted to a cell culture system to circumvent the limitations of mixed cell populations in bulk tissue analysis. However, we did not observe a downregulation in PPARs themselves with KDM6A knockdown; rather, we observed reduction in the protein levels of the transcriptional coactivator PGC-1α, which plays a key role in regulating PPAR signaling (Puigserver and Spiegelman, 2003), as well as mRNA levels of the encoding gene, *PPARGC1A*. PGC-1α protects against kidney disease development (Lynch et al., 2018), is downregulated in UUO kidneys (log_2_ fold change=0.64, *P*=3.75×10^−5^; Dataset 1) and promotes recovery from kidney injury caused by inflammation (Tran et al., 2011). Importantly, PGC-1α has also previously been reported to be repressed by H3K27me3 (Luo et al., 2020) and positively regulated by KDM6A (Zha et al., 2015).

The present study has several limitations that warrant particular emphasis. First, we made an *a priori* decision to study male KDM6A^TubKO^ mice because of a previously described susceptibility to fibrosis in male rodents after UUO (Cho et al., 2012; Metcalfe et al., 2008). However, at the 7-day timepoint chosen, whereas we observed increases in kidney hydroxyproline content and α-SMA immunostaining in UUO mice, we did not detect a significant increase in interstitial fibrosis by Picrosirius Red staining across whole-kidney cross-sections. In retrospect, this observation is unsurprising given recent fate-mapping experiments that have demonstrated that the major collagen-producing cells in the kidney are myofibroblasts that are derived from pericytes and fibroblasts (Kuppe et al., 2021). Accordingly, were matrix deposition to be altered (either increased or decreased) in the kidneys of KDM6A^TubKO^ UUO mice, in comparison to those of wild-type UUO mice, it would likely occur as a secondary (or tertiary) response to changes within tubule epithelial cells, rather than as a primary effect on fibrotic gene production by these cells. Males, however, also express the *Kdm6a* homolog, *Uty*. Thus, the findings in KDM6A^TubKO^ mice reflect those that occur in the absence of KDM6A and presence of UTY. Whether tubule cell knockout of KDM6A in females (i.e. KDM6A and UTY absence) would yield a different phenotype either under normal conditions or in response to injury requires further experimentation. On the one hand, *Kdm6a*^−/−^ embryos die around embryonic day (E)12.5, whereas female *Kdm6a*^−/+^ heterozygotes and male *Kdm6a*^−/Y^ do not, indicating at least some compensatory role for UTY (Shpargel et al., 2012; Tran et al., 2020; Wang et al., 2012a). On the other hand, female *Kdm6a*^−/+^ mice survive through adulthood, whereas male *Kdm6a*^−/Y^ mice die around birth, suggesting a non-compensatory role for UTY in postnatal development (Shpargel et al., 2012; Tran et al., 2020). Likewise, KDM6A has overlapping functions with KDM6B (Manna et al., 2015), and compensation by either or both of KDM6B and UTY may have mitigated the consequences of KDM6A absence. Second, although KDM6A knockdown diminished PGC-1α in cultured HK-2 cells, we submit that our phenotypic observations be viewed through a non-reductionist lens. For instance, we observed an increase in inflammation in UUO kidneys of KDM6A^TubKO^ mice, together with a reduction in tubule cell apoptosis. PPAR signaling can promote programmed cell death (Elrod and Sun, 2008), and PGC-1α has been linked to apoptosis under some conditions (Zhang et al., 2007). However, in other settings, PGC-1α may attenuate apoptosis (Yuan et al., 2021). Thus, it is possible, indeed likely, that KDM6A knockout from tubule cells affects other cellular processes that ultimately manifest with histological evidence of diminished apoptosis under stress conditions. These limitations notwithstanding, there has been significant recent interest in the roles that KDM6A may play in CKD and in the possibility of therapeutically targeting histone modifying enzymes to alter the natural history of kidney disease (Fontecha-Barriuso et al., 2018). The current study adds to a growing body of literature, now describing the (patho)physiological effects of *Kdm6a* deletion from tubule epithelial cells under sham-operated conditions and in the setting of obstructive uropathy.

In summary, in kidney tubule cells, levels of the histone demethylating enzyme *Kdm6a* are both sex and disease dependent. The absence of tubule cell KDM6A in male mice causes relatively subtle transcriptional and phenotypic changes that, under conditions of obstructive uropathy, are characterized by increased kidney inflammation. The present study highlights the limited consequences of deletion of a single histone demethylase from tubule cells *in vivo*. Whether less-specific, broader-acting systemic inhibitor-based strategies to affect epigenetic processes will find a niche for the management of chronic diseases remains to be determined.

## MATERIALS AND METHODS

### *In vivo* study

UUO or sham surgeries were performed in male and female C57BL/6N mice (C57BL/6N/Crl; Charles River Laboratories, Senneville, Quebec, Canada) aged ∼8 weeks (Batchu et al., 2021). Briefly, mice were anesthetized with 2% isoflurane, and an incision was made in the left flank before occlusion of the left ureter using two 5-0 silk sutures. Sham mice underwent the same procedure without ligation of the left ureter. Analgesia was achieved by administering slow-release buprenorphine (0.5 mg/kg subcutaneously) pre-operatively. Mice were followed for 7 or 14 days. *Kdm6a*^fl/fl^ mice (Manna et al., 2015) were provided by Dr Remy Bosselut (Center for Cancer Research, National Cancer Institute, National Institutes of Health, Bethesda, MD, USA) and were bred with *Pax8*-Cre^+^ mice (Bouchard et al., 2004) (stock number 028196, The Jackson Laboratory, Bar Harbor, ME, USA). Male *Pax8*-Cre^+^*Kdma6a*^fl/Y^ mice underwent UUO or sham surgery and were followed for 7 days. Male age-matched sham and UUO *Pax8*-Cre^+^ mice were used as controls. Systolic blood pressure (SBP) was recorded using a CODA non-invasive blood pressure system (Kent Scientific, Torrington, CT, USA), as previously described (Yuen et al., 2012). Plasma TSH was measured by enzyme-linked immunosorbent assay (EKC37924, Biomatik Corp., Kitchener, Ontario, Canada). All experimental procedures adhered to the guidelines of the Canadian Council of Animal Care and were approved by the St. Michael's Hospital Animal Care Committee (ACC888).

### TUNEL

TUNEL staining was performed by members of the Pathology Research Program at Toronto General Hospital (Toronto, Ontario, Canada). TUNEL^+^ nuclei in tubule cells were examined and counted in a masked manner in ten randomly selected fields (100× magnification) in each kidney section.

### RNAscope *in situ* hybridization

RNAscope *in situ* hybridization (Advanced Cell Diagnostics, Hayward, CA, USA) was performed according to the manufacturer's instructions and using custom software, as previously reported (Wang et al., 2012b), with the following probesets: *Ptprc* (318651), *Adgre1* (317961), *Kdm6a* (456961), *Cd86* (403441) and *Cd163* (406631). Hybridization signals were detected using Fast Red, and RNA staining was identified as red puncta on light microscopy. Quantitation of tubule epithelial *Kdm6a* was performed by manually counting red RNAscope puncta within kidney tubule cells in six randomly selected kidney cortical sections (400× magnification) per mouse, in a masked manner. For quantitation of *Ptprc*, *Cd86* and *Cd163*, after *in situ* hybridization, kidney sections were scanned using an Axio Scan.Z1 (Carl Zeiss Microscopy, Jena, Germany) prior to determination of the percentage of positive pixels using the HALO^®^ image analysis platform (Indica Labs, Albuquerque, NM, USA) in five randomly selected cortical areas measuring 300×200 µm from each kidney section.

### Immunohistochemistry

Immunohistochemistry was performed as previously described (Advani et al., 2007) with an anti-α-SMA antibody used at 1:400 dilution [ab5694 (lot GR283004-13), Abcam, Cambridge, MA, USA], an anti-Pax8 antibody at 1:100 dilution [ab191870 (lot GR3398209-8), Abcam] or an anti-cleaved caspase-3 antibody at 1:200 dilution [#9661 (clone D175; lot 45), Cell Signaling Technology, Danvers, MA, USA]. Quantitation of α-SMA immunostaining was performed on digitized images (Axio Scan.z1) using HALO^®^. Cleaved caspase-3^+^ nuclei in kidney tubules were counted in each entire kidney section at 100× magnification by an investigator masked to the study groups.

### Hydroxyproline content

Kidney hydroxyproline content was determined using a Hydroxyproline Assay Kit (Colorimetric) (ab222941, Abcam) and normalized to total protein measured by Quick Start™ Bradford 1× Dye Reagent (5000205, Bio-Rad, Hercules, CA, USA).

### Picrosirius Red staining

After Picrosirius Red staining, kidney sections were digitized (Axio Scan.z1), and the proportional area positively staining red was analyzed using HALO^®^.

### Immunoblotting

Primary kidney tubule epithelial cells were isolated following the protocol described by Ding et al. (2018). In brief, mice were perfused via the left ventricle with 10 ml warm PBS containing 0.5% penicillin–streptomycin, prior to perfusion with 20 ml digestion buffer containing collagenase type II (Worthington Biochemical Corp., Lakewood, NJ, USA) in PBS. Both kidneys were harvested, and the renal capsule and medulla were removed, before mincing and incubation in digestion buffer at 37°C for 5 min. After addition of Dulbecco's modified Eagle medium with 10% fetal bovine serum and 0.5% penicillin–streptomycin, digested kidney tissue was passed through a 70 µm filter, and the filtrate was centrifuged at 50 ***g*** for 5 min. The pellet and supernatant were separated, and the supernatant was further centrifuged at 50 ***g*** for 5 min. Both pellets were combined. Immunoblotting was performed using the following antibodies: anti-KDM6A (UTX) 1:1000 dilution [33510 (clone D3Q1I, lot 2), Cell Signaling Technology], anti-PGC-1α 1:1000 dilution [PA5-72948 (lot: YD3898968A), Thermo Fisher Scientific, Waltham, MA, USA], anti-α-SMA 1:000 dilution [ab5694 (lot GR283004-13), Abcam] and anti-GAPDH 1:1000 dilution [#2188 (clone 14C10, lot 14), Cell Signaling Technology]. The anti-KDM6A antibody used (33510; D3Q1I) is a rabbit monoclonal antibody that was generated by immunizing animals with recombinant protein surrounding Ala490 of the human KDM6A (UTX) protein (Cell Signaling Technology). According to the supplier, the antibody detects full-length KDM6A as well as unidentified protein bands of ∼60-70 kDa. An approximately similar-sized lower-molecular-mass band has previously been described in wild-type, but not KDM6A mutant, *Drosophila* using a different anti-KDM6A antibody (Copur and Müller, 2013). Densitometry was performed using ImageJ version 1.39.

### RNA sequencing

RNA was isolated from kidney homogenates (*n*=5/group) using TRIzol Reagent (Life Technologies, Thermo Fisher Scientific). RNA sequencing was performed using the 6G RNA Sequencing Service (150 bp paired-end, 40 million reads) from ArrayStar (Rockville, MD, USA), as previously described (Batchu et al., 2020). In brief, after quantitation of RNA using a Nanodrop ND-1000, RNA was enriched using oligo (dT) magnetic beads and sequencing libraries were prepared using a KAPA Stranded RNA-Seq Library prep Kit (Illumina, San Diego, CA, USA). Sequencing was performed on an Illumina Novaseq 6000 (150 cycles for both ends). Solexa pipeline v1.8 was used for image analysis. Sequence quality was assessed using FastQC. Hisat2 software was used to align trimmed reads (trimmed 5′, 3′-adaptor bases using cutadapt) to the GRCm38 reference genome (Kim et al., 2015). Transcript abundances were estimated using StringTie (Pertea et al., 2015), and fragments per kilobase of exon per million mapped fragments (FPKM) and differential gene expression were determined with Ballgown (Frazee et al., 2015). Volcano plots were generated, and pathway analysis was performed with the differentially expressed genes in R, Python or shell environment. KEGG pathway analysis was performed for differentially expressed genes, with *P*-values calculated by Fisher's exact test used to estimate the statistical significance of the enrichment of the pathways between groups. Data are deposited to Gene Expression Omnibus (accession number GSE205759).

### HK-2 cells

HK-2 human kidney proximal tubule cells were obtained from American Tissue Type Culture [ATCC; Manassas VA, USA; CRL-2190; mycoplasma not detected and short tandem repeat (STR) authentication by ATCC]. Cells were transfected with a mixture of siRNA directed against KDM6A (sc-76881, Santa Cruz Biotechnology, Dallas, TX, USA) or negative control siRNA (MilliporeSigma, Oakville, Ontario, Canada) at 50 nM concentration with Lipofectamine RNAiMAX Transfection Reagent (Thermo Fisher Scientific) for 6 h before replacement with Keratinocyte SFM medium (Thermo Fisher Scientific). Cells were harvested after 48 h.

### qRT-PCR

RNA was isolated from mouse whole-kidney tissue and HK-2 cells using TRIzol reagent (Thermo Fisher Scientific), and cDNA was reverse transcribed from 0.2 μg (mouse kidney) and 1 μg (HK-2 cells) RNA using a High-Capacity cDNA Reverse Transcription Kit (Thermo Fisher Scientific). Custom-designed primers were from Integrated DNA technologies (Coralville, IA, USA) and had the following sequences: mouse *Ccr2* (forward, 5′-AAAGGAGCCATACCTGTAAATGCC-3′; reverse, 5′-TGCCGTGGATGAACTGAGGTAAC-3′), mouse *Col1a1* (forward, 5′-TTCAGGGAATGCCTGGTGAA-3′; reverse, 5′-ACCTTTGGGACCAGCATCA-3′), mouse *Col1a2* (forward, 5′-GAAAAGGGTCCCTCTGGAGAA-3′; reverse, 5′-AATACCGGGAGCACCAAGAA-3′), mouse *Rpl13a* (forward, 5′-GCTCTCAAGGTTGTTCGGCTGA-3′; reverse, 5′-AGATCTGCTTCTTCTTCCGATA-3′), human *PPARGC1A* (forward, 5′-AGCCTCTTTGCCCAGATCTT-3′; reverse, 5′-GGCAATCCGTCTTCATCCAC-3′), and human *RPL13A* (forward, 5′-TCGTACGCTGTGAAGGCATC-3′; reverse, 5′-TTTTGTGGGGCAGCATACCT-3′). Primers for human *PPARA* (HP226273), *PPARD* (HP209214), *PPARG* (HP226175) and *CCN2* (HP205671) were from OriGene (Rockville, MD, USA).

### Statistics

Data are expressed as mean±s.d. Sample size was determined based on practicability and prior experience with the UUO model (Kaur et al., 2023). Animals were randomly allocated to sham or UUO groups. Analyses of data were performed in a masked manner where feasible. Outlying values were not removed. Statistical analyses were performed using GraphPad Prism 9 for macOS (GraphPad Software Inc., San Diego, CA, USA). Statistical tests were selected after testing for normality and variance inhomogeneity and are stated in the figure legends according to the hypothesis under examination. For multiple comparisons, post hoc testing was only conducted if *F* in ANOVA achieved *P*<0.05. All analyses were two-tailed. *P*<0.05 was considered statistically significant.

## Supplementary Material

10.1242/dmm.049991_sup1 Supplementary information
