# Supplementary material for: Altered expression, but small contribution, of the histone demethylase KDM6A in obstructive uropathy in mice
Source: Dis Model Mech. 2023 Sep 1;16(9):dmm049991. doi: 10.1242/dmm.049991 (PMC10482012; doi:10.1242/dmm.049991)
Supplement: Supplementary information [file dmm-16-049991-s1.pdf]

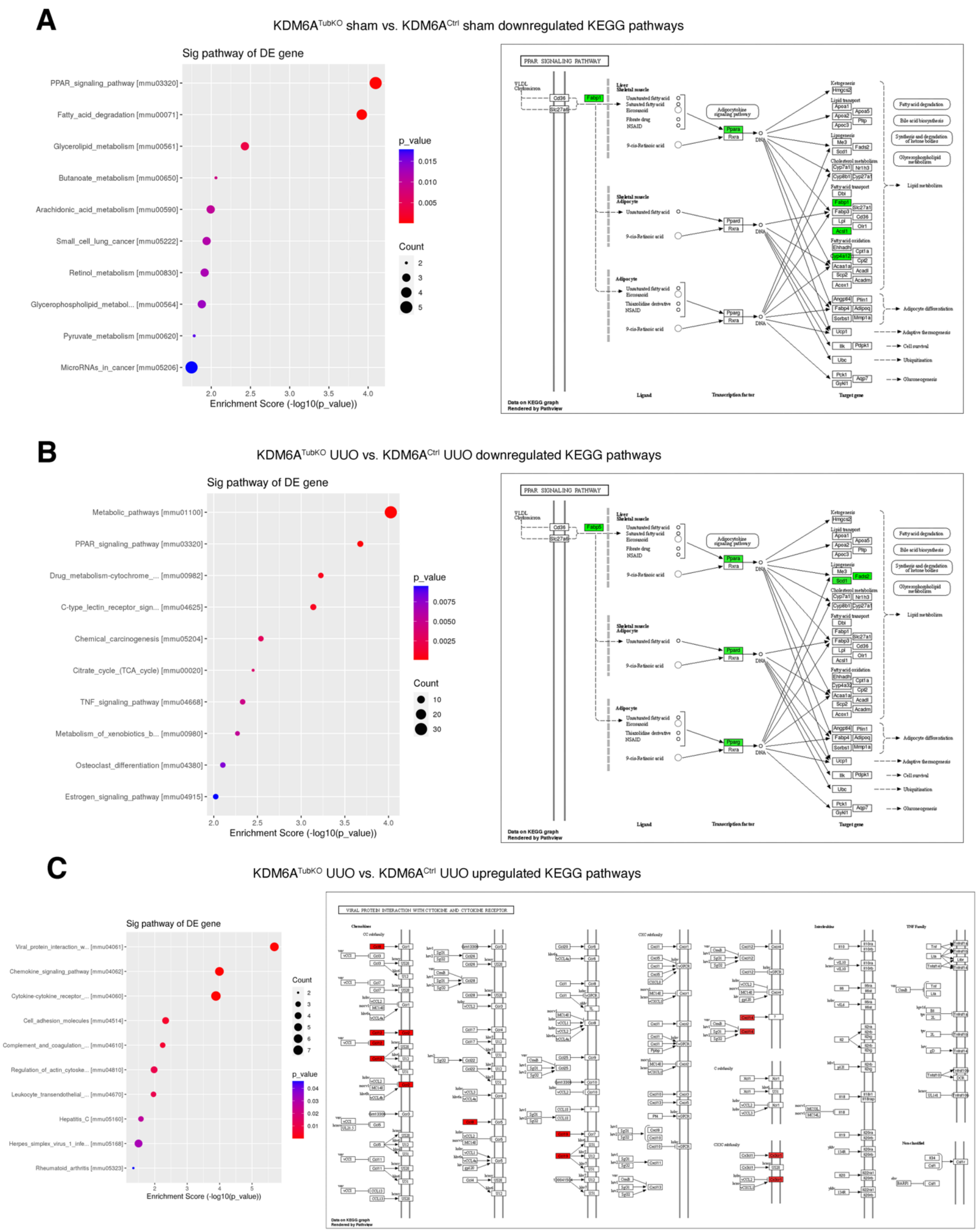
A
KDM6A^TubKO sham vs. KDM6A^Ctrl sham downregulated KEGG pathways
Sig pathway of DE gene
PPAR_signaling_pathway [mmu03320]
Fatty_acid_degradation [mmu00071]
Glycerolipid_metabolism [mmu00561]
Butanoate_metabolism [mmu00650]
Arachidonic_acid_metabolism [mmu00590]
Small_cell_lung_cancer [mmu05222]
Retinol_metabolism [mmu00830]
Glycerophospholipid_metabol... [mmu00564]
Pyruvate_metabolism [mmu00620]
MicroRNAs_in_cancer [mmu05206]
2.0 2.5 3.0 3.5 4.0
Enrichment Score (-log10(p_value))
p_value
0.015
0.010
0.005
Count
2
3
4
5
PPAR SIGNALING PATHWAY
B
KDM6A^TubKO UUO vs. KDM6A^Ctrl UUO downregulated KEGG pathways
Sig pathway of DE gene
Metabolic_pathways [mmu01100]
PPAR_signaling_pathway [mmu03320]
Drug_metabolism-cytochrome_... [mmu00982]
C-type_lectin_receptor_sign... [mmu04625]
Chemical_carcinogenesis [mmu05204]
Citrate_cycle_(TCA_cycle) [mmu00020]
TNF_signaling_pathway [mmu04668]
Metabolism_of_xenobiotics_b... [mmu00980]
Osteoclast_differentiation [mmu04380]
Estrogen_signaling_pathway [mmu04915]
2.0 2.5 3.0 3.5 4.0
Enrichment Score (-log10(p_value))
p_value
0.0075
0.0050
0.0025
Count
10
20
30
PPAR SIGNALING PATHWAY
C
KDM6A^TubKO UUO vs. KDM6A^Ctrl UUO upregulated KEGG pathways
Sig pathway of DE gene
Viral_protein_interaction_w... [mmu04061]
Chemokine_signaling_pathway [mmu04062]
Cytokine-cytokine_receptor_... [mmu04060]
Cell_adhesion_molecules [mmu04514]
Complement_and_coagulation_... [mmu04610]
Regulation_of_actin_cytoske... [mmu04810]
Leukocyte_transendothelial_... [mmu04670]
Hepatitis_C [mmu05160]
Herpes_simplex_virus_1_infe... [mmu05168]
Rheumatoid_arthritis [mmu05323]
2 3 4 5
Enrichment Score (-log10(p_value))
Count
2
3
4
5
6
7
p_value
0.04
0.03
0.02
0.01
VIRAL PROTEIN INTERACTION WITH CYTOKINE AND CYTOKINE RECEPTOR

Fig. S1. Peroxisome proliferator-activated receptor (PPAR) signaling pathways are downregulated with tubule cell *Kdm6a* knockout and associated with upregulation in inflammatory pathways in the kidneys of male mice 7 days after unilateral ureteral obstruction (UUO) surgery. Downregulated KEGG pathways in KDM6A^{TubKO} mice in comparison to KDM6A^{Ctrl} mice after sham surgery (A) or UUO surgery (B). (C) Upregulated KEGG pathways in KDM6A^{TubKO} UUO mice in comparison to KDM6A^{Ctrl} mice. The images on the left show dotplots for the top 10 pathways based on enrichment score (-log10(p_value)) for each comparison. The images on the right show the pathway maps for PPAR_signaling_pathway (mmu03320) (A and B) or Viral_protein_interaction_with_cytokine_and_cytokine_receptor (mmu04061) (C). mmu03320 is significantly downregulated in the comparisons of KDM6A^{TubKO} sham vs. KDM6A^{Ctrl} sham and KDM6A^{TubKO} UUO vs. KDM6A^{Ctrl} UUO, whereas mmu04061 is significantly upregulated in KDM6A^{TubKO} UUO vs. KDM6A^{Ctrl} UUO.

Table S1. Metabolic characteristics of KDM6A^{Ctrl} and KDM6A^{TubKO} mice 7 days after sham surgery or unilateral ureteral obstruction (UUO).

	KDM6A^{Ctrl} sham	KDM6A^{Ctrl} UUO	KDM6A^{TubKO} sham	KDM6A^{TubKO} UUO
n	6	9	7	8
Body weight (g)	23.5±2.0	25.0±1.6	23.4±2.1	24.3±1.5
Left kidney weight (g)	0.133±0.021	0.208±0.045[a]	0.124±0.025[b]	0.193±0.029[cd]
Left kidney weight:body weight (%)	0.56±0.07	0.83±0.18[a]	0.53±0.09[b]	0.80±0.13[cd]
Systolic blood pressure (mmHg)	108±15	116±19	100±12	106±13

Values are mean ± S.D.. [a]*P* < 0.01 vs KDM6A^{Ctrl} sham, [b]*P* < 0.001 vs. KDM6A^{Ctrl} UUO, [c]*P* < 0.05 vs. KDM6A^{Ctrl} sham, [d]*P* < 0.01 vs. KDM6A^{TubKO} sham by one-way ANOVA followed by Tukey's post hoc test.

Table S2. Top 5 significantly enriched KEGG pathways in the kidneys of $KDM6A^{Ctrl}$ mice 7 days after unilateral ureteral obstruction (UUO) surgery in comparison to sham-operated $KDM6A^{Ctrl}$ mice.

ID	Term	Count	p_value	FDR	Enrichment_Score
Upregulated					
mmu04380	Osteoclast_differentiation	75	3.83E-19	6.93E-17	18.42
mmu04668	TNF_signaling_pathway	69	4.36E-19	6.93E-17	18.36
mmu05166	Human_T-cell_leukemia_virus_1_infection	116	2.69E-18	2.85E-16	17.57
mmu05200	Pathways_in_cancer	200	1.01E-15	8.06E-15	14.99
mmu04064	NF-kappa_B_signaling_pathway	62	1.86E-15	1.82E-13	14.73
Downregulated					
mmu01100	Metabolic_pathways	335	1.78E-104	5.27E-102	103.75
mmu00280	Valine_leucine_and_isoleucine_degradation	37	1.04E-29	1.62E-27	28.96
mmu01200	Carbon_metabolism	48	1.24E-24	1.21E-22	23.91
mmu04146	Peroxisome	40	2.46E-24	1.81E-22	23.61
mmu00071	Fatty_acid_degradation	30	1.92E-22	1.13E-20	21.72

Count = count of the differentially expressed genes directly associated with the listed pathway
p_value = enrichment p=value of the PathwayID using Fisher's exact test
FDR = false discovery rate of the PathwayID
Enrichment score = enrichment value of the PathwayID, equals "-log10(p_value)"

Disease Models & Mechanisms • Supplementary information

Table S3. Top 5 significantly enriched KEGG pathways in the kidneys of KDM6A^{TubKO} mice 7 days after unilateral ureteral obstruction (UUO) surgery in comparison to sham-operated KDM6A^{TubKO} mice.

ID	Term	Count	p_value	FDR	Enrichment_Score
Upregulated					
mmu04380	Osteoclast_differentiation	79	4.89E-20	1.57E-17	19.31
mmu04668	TNF_signaling_pathway	72	1.63E-19	2.62E-17	18.79
mmu05166	Human_T-cell_leukemia_virus_1_infection	121	3.12E-18	3.33E-16	17.51
mmu05200	Pathways_in_cancer	216	1.44E-17	1.15E-15	16.84
mmu04210	Apoptosis	78	3.89E-17	2.49E-15	16.41
Downregulated					
mmu01100	Metabolic_pathways	398	4.09E-125	1.26E-122	124.39
mmu01200	Carbon_metabolism	60	7.35E-33	1.14E-30	32.13
mmu00280	Valine_leucine_and isoleucine_degradation	37	7.25E-27	7.47E-25	26.14
mmu04146	Peroxisome	43	1.47E-24	1.13E-22	23.83
mmu00190	Oxidative_phosphorylation	51	2.70E-22	1.67E-20	21.57

Count = count of the differentially expressed genes directly associated with the listed pathway
p_value = enrichment p=value of the PathwayID using Fisher’s exact test
FDR = false discovery rate of the PathwayID
Enrichment score = enrichment value of the PathwayID, equals “-log10(p_value)”

Table S4. Top 5 significantly enriched KEGG pathways in the kidneys of KDM6A^{TubKO} mice 7 days after sham surgery in comparison to sham-operated KDM6A^{Ctrl} mice.

ID	Term	Count	p_value	FDR	Enrichment_Score
Upregulated					
mmu01100	Metabolic_pathways	23	3.22E-07	3.34E-05	6.49
mmu00260	Glycine_serine_and_threonine_metabolism	4	4.59E-05	0.00238	4.34
mmu05143	African_trypanosomiasis	3	0.00098	0.03383	3.01
mmu00330	Arginine_and_proline_metabolism	3	0.00251	0.05630	2.60
mmu05144	Malaria	3	0.00293	0.05630	2.49
Downregulated					
mmu03320	PPAR_signaling_pathway	5	8.01E-05	0.00789	4.10
mmu00071	Fatty_acid_degradation	4	0.00012	0.00789	3.92
mmu00561	Glycerolipid_metabolism	3	0.00372	0.16261	2.43
mmu00650	Butanoate_metabolism	2	0.00871	0.21613	2.06
mmu00590	Arachidonic_acid_metabolism	3	0.01017	0.21613	1.99

Count = count of the differentially expressed genes directly associated with the listed pathway
p_value = enrichment p=value of the PathwayID using Fisher’s exact test
FDR = false discovery rate of the PathwayID
Enrichment score = enrichment value of the PathwayID, equals “-log10(p_value)”

Disease Models & Mechanisms • Supplementary information

Table S5. Top 5 significantly enriched KEGG pathways in the kidneys of KDM6A^{TubKO} mice 7 days after unilateral ureteral obstruction (UUO) surgery in comparison to KDM6A^{Ctrl} mice after UUO surgery.

ID	Term	Count	p_value	FDR	Enrichment_Score
Upregulated					
mmu04061	Viral_protein_interaction_with_cytokine_receptor	6	2.05E-06	0.00019	5.69
mmu04062	Chemokine_signaling_pathway	6	0.00010	0.00395	3.99
mmu04060	Cytokine_cytokine_receptor_interaction	7	0.00013	0.00395	3.89
mmu04514	Cell_adhesion_molecules	4	0.00463	0.10369	2.33
mmu04610	Complement_and_coagulation_cascades	3	0.00569	0.10369	2.24
Downregulated					
mmu01100	Metabolic_pathways	30	9.42E-05	0.02157	4.03
mmu03320	PPAR_signaling_pathway	6	0.00021	0.02410	3.68
mmu00982	Drug_metabolism-cytochrome_P450	5	0.00059	0.04156	3.23
mmu04625	C-type_lectin_receptor_signaling_pathway	6	0.00073	0.04156	3.14
mmu055204	Chemical_carcinogenesis	5	0.00289	0.13220	2.54

Count = count of the differentially expressed genes directly associated with the listed pathway
p_value = enrichment p=value of the PathwayID using Fisher's exact test
FDR = false discovery rate of the PathwayID
Enrichment score = enrichment value of the PathwayID, equals "-log10(p_value)"

Disease Models & Mechanisms • Supplementary information

Table S6. Metabolic characteristics of male and female C57BL/6N mice 14 days after sham surgery or unilateral ureteral obstruction (UUO).

	Male sham	Female sham	Male UUO	Female UUO
n	6	6	6	6
Body weight (g)	24.0±1.7	19.8±1.2[a]	22.2±2.3	19.0±0.9[bc]
Left kidney weight (g)	0.137±0.009	0.114±0.007	0.249±0.054[df]	0.201±0.034[eg]
Left kidney weight:body weight (%)	0.57±0.02	0.57±0.01	1.13±0.24[df]	1.06±0.14[df]

Values are mean ± S.D.. [a]$P < 0.01$ vs male sham, [b]$P < 0.001$ vs. male sham, [c]$P < 0.05$ vs. male UUO, [d]$P < 0.0001$ vs. male sham, [e]$P < 0.05$ vs. male sham, [f]$P < 0.0001$ vs. female sham, [g]$P < 0.001$ vs. female sham, [h]$P < 0.0001$ vs. male sham by one-way ANOVA followed by Tukey's post hoc test.

Dataset 1. Differentially expressed genes in the kidneys of KDM6A^{Ctrl} and KDM6A^{TubKO} mice 7 days after sham or unilateral ureteral obstruction surgery.

Click here to download Dataset 1

Dataset 2. Significantly enriched KEGG pathways in the kidneys of KDM6A^{Ctrl} and KDM6A^{TubKO} mice 7 days after sham or unilateral ureteral obstruction surgery.

Click here to download Dataset 2

Disease Models & Mechanisms • Supplementary information
